# Soft tissue mechanosensing and remodeling: the core regulatory role of Piezo/TRPV4 ion channels

**DOI:** 10.3389/fcell.2026.1869360

**Published:** 2026-08-18

**Authors:** Xuanqiang Fan, Mingwei Shi, Zhen Wang, Chi Zhao, Yifan Zhao, Yunfeng Zhou, Hui Xu

**Affiliations:** 1 The Third Affiliated Hospital of Henan University of Chinese Medicine, Zhengzhou, China; 2 School of Acupuncture-Moxibustion and Tuina, Henan University of Chinese Medicine, Zhengzhou, China

**Keywords:** extracellular matrix stiffening, mechanical therapeutic window, Piezo1, soft tissue mechanosensing, TRPV4

## Abstract

Soft tissue fibrosis and chronic pain involve a cycle linking extracellular matrix (ECM) stiffening, inflammation, and neural sensitization. This review develops a multiscale framework that spans from molecular mechanisms to organ-level physiology and focuses on the directly mechanosensitive Piezo1/2 channels and the mechano-responsive TRPV4 channel, drawing on their distinct molecular architectures to ground divergent gating properties. We examine how these channels convert membrane tension and integrin-mediated traction into Ca^2+^ and other ionic signals that, together with Ca^2+^-independent pathways engage YAP/TAZ, NF-κB, and TGF-β pathways, thereby reshaping transcriptional programs. We then conceptualize healthy ECM as a “spatial filter,” noting that viscoelasticity and non-affine fiber rearrangements buffer superficial loads, whereas fibrotic cross-linking erodes this protection, chronically exposing fibroblasts and sensory endings to elevated mechanical and inflammatory cues. This may drive pathological “stiffness–inflammation–fibrosis/pain” loops. Finally, we propose a mechanical therapeutic window in which mechanical interventions, parameterized by channel modulation and/or viscoelastic biomaterials, reset the local environment toward a near-physiological state. Within this Goldilocks zone, reparative and inflammation-resolving pathways can be preferentially engaged while minimizing damage and pain amplification. This integrated perspective offers a conceptual framework for understanding fibrosis-associated disorders and chronic soft-tissue pain, and may inform therapies targeting Piezo/TRPV4 signaling and ECM viscoelasticity.

## 1 Introduction

Soft tissues are widely distributed throughout the human body, from skin, fascia, and tendons to visceral interstitium. They not only bear loads, buffer impacts, and transmit mechanical signals, but also participate deeply in inflammatory responses, immune homeostasis, and metabolic regulation. In mechanical terms, most soft tissues span a stiffness range of approximately 0.1–100 kPa under quasi-static loading, in marked contrast to mineralized tissues such as bone (Discher et al., 2005; Swift et al., 2013). Compared with high-stiffness tissues such as bone, most soft tissues are, in material terms, closer to a porous viscoelastic composite consisting of collagen fiber networks, proteoglycan-rich matrices, and interstitial fluid. They exhibit stress relaxation, creep, frequency-dependent stiffness, and marked energy dissipation (Chaudhuri et al., 2020). These material properties mean that external loads, as they propagate from the surface toward deeper layers, undergo substantial attenuation in magnitude and remodeling in temporal structure. This generates a physiologically meaningful spatiotemporal mechanical filtering mechanism: on the one hand, buffering and reducing transient peak mechanical inputs to prevent deep cells from being exposed to excessively high and rapid mechanical perturbations (Chaudhuri et al., 2020; Courbot and Elosegui-Artola, 2025); on the other hand, preserving sensitivity to long-term stress redistribution and matrix remodeling, thereby providing an essential mechanical input window for tissue homeostatic regulation (Courbot and Elosegui-Artola, 2025; Wu et al., 2025).

At the cellular scale, traditional views have emphasized the role of structures such as integrins, the cytoskeleton, and focal adhesions in sensing and transducing mechanical signals. Over the past decade, the discovery and characterization of mechanosensitive ion channels such as the Piezo family and TRPV4 have enabled an understanding of the “force-electric--chemical” conversion process at the molecular and even atomic levels. Piezo1/2 have been shown to be core components of mechanically activated cation currents in many cell types (Table 1). Their gating depends on membrane tension and cytoskeletal pulling transmitted by the molecular clutch; cryo-electron microscopy structures and theoretical models have revealed the mechanisms by which membrane tension is transferred into channel conformational changes (Zhang et al., 2016; Saotome et al., 2018). TRPV4, a polymodal transient receptor potential (TRP) channel, is widely expressed in fibroblasts, endothelial cells, and immune cells. By integrating stretch, osmotic pressure, and lipid ligands, it serves as a major Ca^2+^ entry route linking mechanical stimulation with inflammatory amplification and fibrotic remodeling (Adapala et al., 2021). However, it should be noted that whether TRPV4 is directly gated by mechanical force or indirectly modulated through mechano-chemical coupling remains an active area of investigation. Local Ca^2+^ microdomains and oscillatory signals mediated by these channels are integrated into transcriptional and epigenetic networks through downstream axes including YAP/TAZ, NF-κB, and TGF-β/Smad, thereby contributing to cell phenotypes associated with highly synthetic, highly inflammatory, and highly contractile states (Michalick and Kuebler, 2020; Adapala et al., 2021).

**TABLE 1:** Comparative expression and properties of Piezo1, Piezo2, and TRPV4 across key cell types relevant to soft tissue mechanobiology.

Channel	Cell type	Expression	Proposed function	Evidence	References
Piezo1	Fibroblasts	Upregulated in fibrotic skin, kidney, lung, and liver	ECM stiffness sensing; myofibroblast activation and ECM deposition in fibrosis	Strong	Fu et al. (2021), Yao et al. (2022), He et al. (2024), Dong L. et al. (2025), Rashidi et al. (2025)
Piezo1	Macrophages	Expressed; upregulated in fibrotic liver	Context-dependent polarization: pro-inflammatory/pro-fibrotic via Notch/NF-kB/STAT; pro-resolving via efferocytosis	Moderate	Luo S. et al. (2023), Wang Y. et al. (2024)
Piezo1	Endothelial cells	Expressed in Schlemm canal and other endothelial populations	ECM stiffness sensing; stiffness-dependent YAP/TAZ activation and remodeling	Moderate	Li H. et al. (2024), M et al. (2025)
Piezo1	Sensory neurons	Low expression relative to Piezo2	Minor role in mechanical nociception	Limited	Wan et al. (2024)
Piezo2	Sensory neurons (DRG)	Highly expressed in DRG neurons; enriched in low-threshold mechanoreceptors and a subset of nociceptors	Low-threshold touch transduction; mechanical pain in inflammatory and neuropathic contexts	Strong	Sánchez-Carranza et al. (2024), Habib et al. (2025), Cheng et al. (2026), Mulhall et al. (2026)
Piezo2	Fibroblasts	Not detectably expressed	No established role	Speculative	-
TRPV4	Fibroblasts	Expressed; upregulated by TGF-beta1	Mechano-chemical integration; synergizes with TGF-beta to promote myofibroblast differentiation and fibrosis	Strong	Rahaman et al. (2014), Sharma et al. (2019)
TRPV4	Macrophages	Expressed	Context-dependent: pro-inflammatory/pro-fibrotic vs. anti-inflammatory; regulates NF-kB and inflammasome	Moderate	Scheraga et al. (2016)
TRPV4	Endothelial cells	Highly expressed	Barrier function, vascular permeability, and endothelial-to-mesenchymal transition (EndMT)	Strong	Mukherjee et al. (2025)
TRPV4	Sensory neurons	Expressed in sensory neurons and Schwann cells	Mechanical allodynia; inflammatory pain sensitization; Schwann cell-mediated mechanical nociception	Moderate	Mulpuri et al. (2025), Ruviaro et al. (2025)

Evidence strength: Strong = replicated in multiple independent models with genetic and pharmacological validation; Moderate = supported by multiple studies but context-dependent or lacking full genetic validation across organs; Limited = preliminary or single-study evidence; Speculative = inferred or suggested without direct experimental support. a Piezo1 has been shown to be force-dependently recruited to integrin-based focal adhesions (FAs) where it colocalizes with talin and vinculin (Yao et al., 2022). Functional coupling has been demonstrated by imaging and Ca2+ imaging, but direct atomic-level binding between Piezo1 intracellular domains and talin or vinculin has not been confirmed structurally.

At the tissue and organ levels, when the extracellular matrix (ECM) undergoes pathological remodeling characterized by increased collagen deposition, cross-linking, and reduced stress-relaxation capacity,we propose that the originally protective “spatial filter” may progressively fail. Under similar macroscopic strain, evidence from fibrotic models suggests that deeper regions then experience higher internal stress peaks and shorter buffering times, effectively shifting the operating point of mechanosensitive ion channels upward and making Piezo1/Piezo2/TRPV4 easier to activate (Lin et al., 2025; Chen et al., 2026). These pathological mechanical changes are thought to sustain the multicellular positive-feedback loops, which link ECM stiffening, fibroblast activation, immune amplification, and neural sensitization, and that are examined in detail in Chapters 4 and 5 (Gromakovskis, 2025; Lin et al., 2025).

Against this background, a systematic analysis of Piezo/TRPV4 in soft tissue mechanosensing and remodeling is essential for understanding the multilayered nature of stiffness–inflammation–fibrosis/pain loops, and for the development of targeted interventions. Chapter 2 starts from gating thermodynamics and the two physical models of “force-from-lipids” and “force-from-tether” to dissect the mechanical gating mechanisms of Piezo/TRPV4 and the “frequency decoding” logic of Ca^2+^ oscillations. Chapter 3 introduces the concept of a “porous viscoelastic spatial filter” to clarify differences between healthy and pathological ECM in depth-dependent mechanical attenuation. Chapters 4 and 5, respectively, focus on fibroblast–immune and sensory neuron–immune networks to examine how Piezo1/TRPV4 may promote structural collapse and stiffening, and how they may contribute to neural sensitization and pain in stiffened environments. Chapter 6 then discusses, on the basis of these loops, the mechanobiological rationale of channel inhibitors, viscoelastic hydrogels, massage/pressure, and extracorporeal shock wave therapy (ESWT), and proposes the concept of a “mechanical therapeutic window” as a framework for future individualized, multimodal intervention strategies.

## 2 Physical gating mechanisms of mechanosensitive ion channels and mechanical matching in soft tissues

**FIGURE 1:**
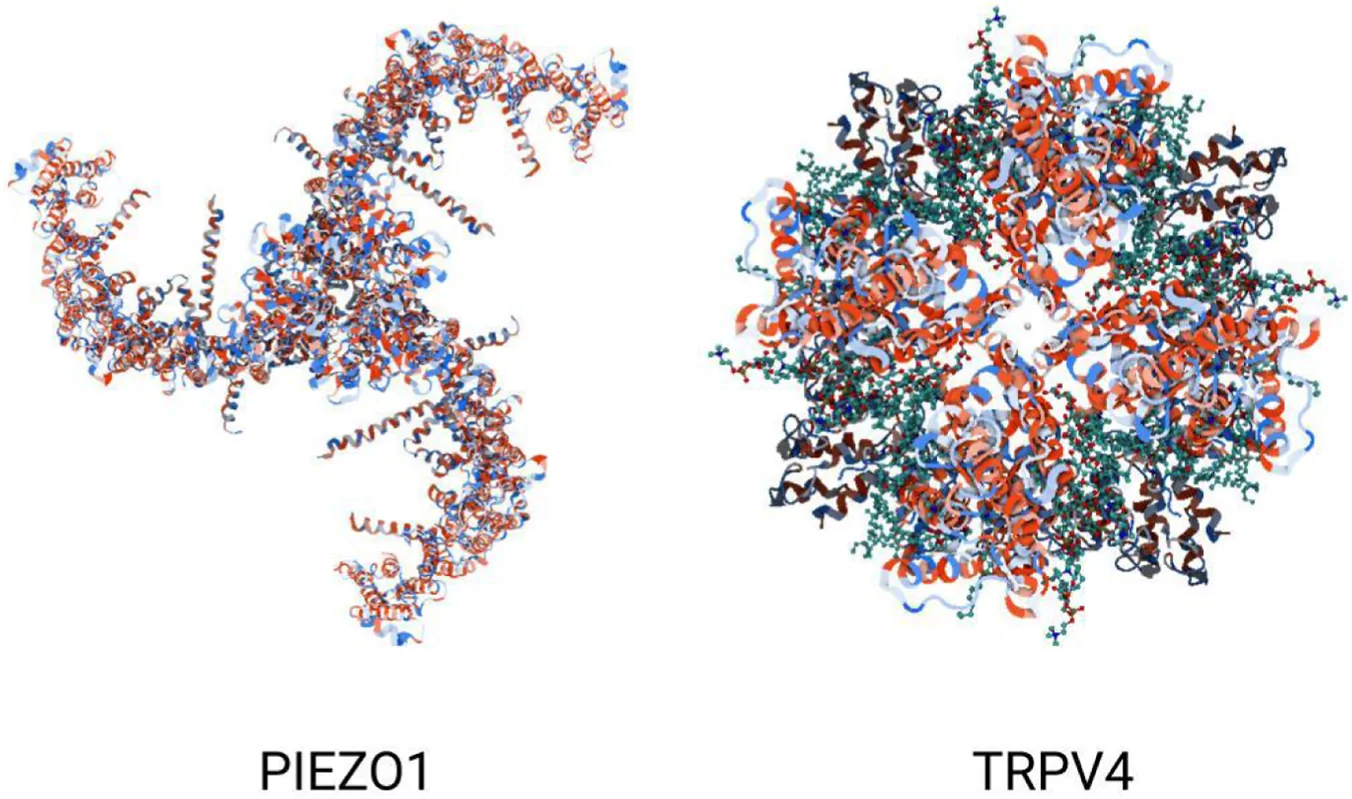
Ribbon representation of mouse Piezo1 (left; PDB: 6B3R) and full-length human TRPV4 in the apo state (right; PDB: 8T1B). Piezo1 assembles as a ∼900 kDa homotrimer adopting a three-bladed propeller architecture. TRPV4 assembles as a ∼400 kDa homotetramer: each subunit contains six transmembrane helices. Created in BioRender. w, W. (2026) https://BioRender.com/0zao7r3.

**FIGURE 2:**
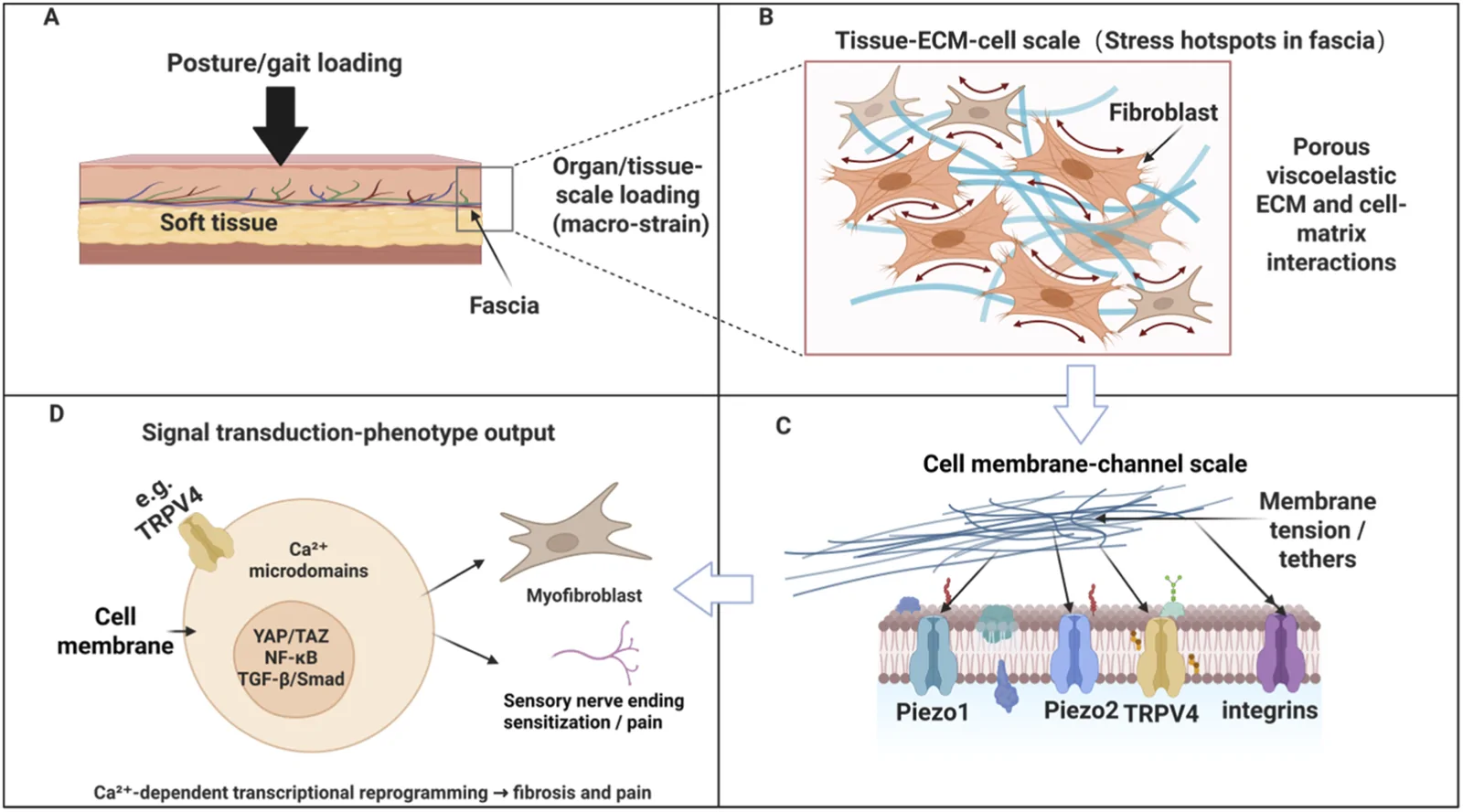
Multiscale framework of soft tissue mechanosensing centered on Piezo/TRPV4. **(A)** Posture/gait-related loading is illustrated by downward arrows applied to superficial soft tissues, with the fascia layer highlighted as the region of interest. **(B)** Zoom-in of fascia showing fibroblasts embedded in a porous, viscoelastic extracellular matrix (ECM); red double-headed arrows indicate local stress hotspots arising from cell–matrix interactions. **(C)** Mechanical tension from ECM fibers is transmitted to the cell membrane via tethers, acting on integrins and mechanosensitive channels. **(D)** Channel opening generates Ca^2+^ microdomains that activate YAP/TAZ, NF-κB and TGF-β/Smad signaling, driving myofibroblast differentiation (α-SMA stress fibers) and sensitization of sensory nerve endings, ultimately contributing to fibrosis and pain. Created in BioRender. w, W. (2026) https://BioRender.com/k0qkudm.

Mechanosensitive ion channels are core molecular elements of cellular mechanotransduction; their gating properties largely determine how cells sense and encode physical signals originating from both the external environment and their own activity (Zhang et al., 2016). In the low-stiffness, porous viscoelastic environment characteristic of soft tissues, Piezo1/2 and TRPV4 exhibit relatively distinct activation patterns: the Piezo family responds more directly to changes in membrane tension, whereas TRPV4 depends on multimodal mechano-chemical coupling involving integrins, the cytoskeleton, and osmotic forces. Through different physical conduction routes, these channels convert mechanical stimuli into changes in membrane potential and Ca^2+^ influx, ultimately shaping downstream signaling network reprogramming (Figures 1, 2).

### 2.1 Gating thermodynamics and two-core physical conduction models

The distinct gating properties of Piezo1 and TRPV4 (Table 2) originate from their fundamentally different molecular architectures (Figure 1). Piezo1 assembles as a ∼900 kDa homotrimer that adopts a three-bladed propeller-like conformation, with each subunit contributing 38 transmembrane helices (Saotome et al., 2018). The distal blades reside within the plane of the lipid bilayer and function as the primary membrane-tension-sensing elements; force is transmitted from blade to central pore via the beam helix, a long α-helical segment that mechanically couples peripheral blade displacement to pore opening (Saotome et al., 2018). On the intracellular face, N-terminal THU1/2 domains and a cytosolic plug domain provide candidate interaction interfaces for talin- and vinculin-mediated force transmission (Shan et al., 2025), offering a structural basis for the tether model of Piezo1 gating.

**TABLE 2:** Comparative biophysical and gating properties of Piezo1 and TRPV4.

Property	Piezo1	TRPV4	Key references
Oligomeric state	Homotrimer (∼900 kDa)	Homotetramer (∼400 kDa)	Saotome et al. (2018); Shan et al. (2025); Plant and Strotmann (2007)
TM helices per subunit	38	6	Saotome et al. (2018); Plant and Strotmann (2007)
Direct mechanosensitivity (lipid bilayer reconstitution)	Yes	No (requires PLA_2_/second messengers)	Syeda et al. (2016); Saotome et al. (2018); Vriens et al. (2004)
Primary gating model	Force-from-lipids + Force-from-tether	Force-from-tether (integrin–cytoskeleton dependent)	Wiggins and Phillips (2004); Lacroix and Wijerathne (2025); Rahaman et al. (2014); Sharma et al. (2019)
Activation stimulus	Membrane tension (primary); additional modalities under investigation	Multimodal: stretch, hypotonicity, temperature lipid ligands (AA metabolites)	Coste et al. (2010); Lacroix and Wijerathne (2025); Plant and Strotmann (2007); Watanabe et al. (2003)
Inactivation kinetics	Fast (∼16 ms whole-cell; ∼45 ms patch); biphasic	Slow; sustained Ca^2+^ elevation; Ca^2+^-dependent inactivation	Coste et al. (2010); Gottlieb et al. (2012); Watanabe et al. (2002); Watanabe et al. (2003)
Single-channel conductance	∼35 pS (monovalent, physiological divalents); ∼15 pS (Ca^2+^); ∼68 pS (divalent-free)	∼30–90 pS (voltage-dependent; ∼30 pS inward/∼90 pS outward)	Gottlieb et al. (2012); Gnanasambandam et al. (2015); Strotmann et al. (2000)
Ca^2+^ selectivity (P_Ca/P_Na)	Non-selective cation channel	∼6–10 (moderate Ca^2+^ selectivity; WT ∼6.9)	Gnanasambandam et al. (2015); Voets et al. (2002)
Force-dependent subcellular enrichment	Clusters at high-tension focal adhesions	Focal adhesions + cell–cell junctions + primary cilia	Yao et al. (2022); Verkest et al. (2025); Sharma et al. (2019); Sokabe et al. (2010)
Ca^2+^ signal signature	Local Ca^2+^ flickers at adhesions	Global, sustained Ca^2+^ elevation	Ellefsen et al. (2019); Rahaman et al. (2014)
Pharmacological tools	Yoda1 (agonist), GsMTx4 (inhibitor), Dooku1 (antagonist)	GSK1016790A (agonist), GSK2193874 (antagonist), HC-067047 (antagonist)	Bae et al. (2011); Cheung et al. (2017); Thorneloe et al. (2008); Thorneloe et al. (2012)

All biophysical parameters are sourced from original discovery and characterization papers alongside confirmatory structural and functional studies. Single-channel conductance and inactivation kinetics represent consensus ranges that vary with cell type, recording configuration, and ionic conditions. The absence of TRPV4 direct mechanosensitivity in minimal reconstituted systems is based on negative evidence and remains an active area of investigation (see Section 2.1). A vertical bar (|) in the Key references column separates Piezo1 (left) and TRPV4 (right) citations.

TRPV4 assembles as a ∼400 kDa homotetramer. A recent cryo-EM structure of full-length human TRPV4 resolved at 3.0 Å(Nadezhdin et al., 2023)confirms the canonical TRP channel fold: each subunit contains six transmembrane helices, with S1–S4 forming a peripheral sensor domain and S5–S6 together with the intervening pore-loop constituting the central ion-conduction pathway. A large N-terminal ankyrin repeat domain (ARD) extends into the cytoplasm, where it mediates coupling to the integrin–cytoskeleton network (Sharma et al., 2019). Unlike Piezo1, whose expansive blade architecture enables direct detection of membrane tension changes (force-from-lipids), TRPV4 exhibits a compact transmembrane fold that is consistent with its predominant dependence on the tether pathway for mechanical activation. Notably, the Nadezhdin et al. structure also captured TRPV4 in complex with the small GTPase RhoA, providing direct structural evidence for TRPV4 coupling to cytoskeleton-regulatory proteins. These architectural differences provide a structural framework for the two canonical force-transmission models introduced below.

From an energetic perspective, the opening and closing of mechanosensitive ion channels can be abstracted as a two-state thermodynamic process. The core question is how mechanical force alters the conformational free energy of the channel protein, enabling it to cross the energy barrier and transition from the closed state to the open state. A commonly used simplified model is:

$$\Delta \text{Gopen}‐\text{closed}=\Delta G_{0}‐\gamma \sigma$$

where σ denotes membrane tension or an equivalent tensile force along a tether, γ is the force–conformation coupling coefficient, ΔG_open-closed is the free-energy difference between the open and closed states, and ΔG_0_ is the intrinsic free-energy difference in the absence of external mechanical force. As σ increases, ΔG_open-closed decreases, and the channel opening probability increases exponentially according to the Boltzmann distribution (Wiggins and Phillips, 2004). Within this framework, contemporary mechanobiology commonly employs two complementary mechanical force-transmission models to understand channel gating: the lipid-membrane tension model (“force-from-lipids”) and the tether model (“force-from-tether”). These mechanisms are not mutually exclusive but act with different weights under different tissue scales and microenvironmental conditions.

The lipid-membrane tension model posits that mechanical force first alters physical parameters of the plasma membrane, including lateral tension, spontaneous curvature, and thickness, and then couples to transmembrane domains of the channel via changes in the lateral pressure profile of the lipid bilayer, thereby modifying its conformational free energy (Wiggins and Phillips, 2004). When a cell membrane is stretched, local lateral tension rises, and originally dome-shaped or invaginated membrane regions tend to flatten. This geometric and energetic change generates additional tensile stress in the region where the channel is embedded, lowering the free energy of certain conformational states and increasing the occupancy probability of the open state. This model explains well the high sensitivity of the Piezo family to membrane tension changes observed in artificial lipid bilayers, membrane patches, and whole-cell stretch experiments (Wiggins and Phillips, 2004; Zhang et al., 2016; Saotome et al., 2018).

In dense soft tissues such as tendons, fascia, and deep skeletal muscle, macroscopic loads undergo substantial attenuation and redistribution within the ECM scaffold before reaching the cell surface. Simple global membrane stretch therefore cannot fully explain how cells finely sense small variations in ECM stiffness and traction. Instead, the tether model provides a clearer biomechanical explanation: mechanical signals are anchored via integrins and focal adhesion complexes, which connect to intracellular F-actin to form a force-transmission chain. The core of this chain is the molecular clutch: integrins and adhesion proteins such as talin and vinculin dynamically bind F-actin, partially arresting retrograde actin flow, so that myosin-II-generated traction accumulates as stable tension at the cell-matrix interface (Elosegui-Artola et al., 2016; Sun et al., 2016). This tension, transmitted through cytoskeleton–membrane coupling, acts on mechanosensitive channels and shifts their conformational free-energy baseline. Accordingly, in dense soft tissues, the mechanical forces perceived by cells are better described as local cytoskeletal tension and adhesion-interface stress, rather than as simple global membrane stretch. Overall, Piezo1 in soft tissues is typically regulated by both lipid-membrane tension and tether-mediated pulling forces, whereas the mechanical gating of TRPV4 is more dependent on the tether pathway formed by integrins, focal adhesions, and the cytoskeleton. Under physiological conditions, these two models often exhibit pronounced spatiotemporal synergy. Yao et al. demonstrated that Piezo1 is specifically recruited to mature focal adhesion microdomains, in a force-and cell state-dependent manner, where it triggers highly localized Ca^2+^ influx events that rapidly convert local cytoskeletal tension into biochemical signals (Yao et al., 2022). These biophysical differences carry important functional consequences for the types of mechanical stimuli each channel preferentially encodes in soft tissues (Table 2).

Consistent with its tether-dependent gating (Table 2), TRPV4 is functionally coupled to the integrin–cytoskeleton system: its activation is required for matrix stiffness-induced YAP/TAZ nuclear translocation and downstream EMT programs, and its genetic deletion or pharmacological inhibition abrogates stiffness-dependent fibrotic responses (Sharma et al., 2019). At the same time, TRPV4 integrates mechanical cues with biochemical signals triggered by pro-fibrotic factors such as TGF-β1, making it an important “mechanical–biochemical integration node” in soft tissue fibrosis (Sun et al., 2016; Sharma et al., 2019). In addition to Ca^2+^, TRPV4 conducts Na^+^ with a reported P_Ca/P_Na ratio of ∼6–10 (Voets et al., 2002). Under physiological ionic gradients, a substantial fraction of the TRPV4-mediated inward current is carried by Na^+^, which can depolarize the plasma membrane and secondarily engage voltage-gated Ca^2+^ channels in excitable cells or modulate Na^+^/Ca^2+^ exchanger activity. These Na^+^-dependent effects may contribute to TRPV4’s roles in cell volume regulation and vascular tone independently of its Ca^2+^ conductance.

It is important to note, however, that whether TRPV4 functions as a directly mechanosensitive channel—in the sense that membrane tension alone suffices to gate the channel in the absence of second messengers—remains an active area of investigation. Unlike Piezo1, which has been shown to be inherently mechanosensitive when reconstituted in artificial lipid bilayer systems (Saotome et al., 2018), direct evidence for TRPV4 mechanosensitivity in minimal reconstituted systems is currently lacking. Several studies indicate that TRPV4 activation by mechanical stimuli in cells requires intact integrin signaling, phospholipase A_2_ activity, and the generation of arachidonic acid metabolites (Vriens et al., 2004; Plant and Strotmann, 2007). Vriens et al. demonstrated that PLA_2_ inhibitors abolish TRPV4 activation by hypotonic swelling, whereas heat and 4α-PDD activate TRPV4 through a distinct, PLA_2_-independent pathway, indicating that at least one major form of TRPV4 mechano-activation is mediated indirectly through ligand-dependent routes rather than through direct force-to-channel coupling. Throughout this review, we therefore refer to TRPV4 as a “mechano-responsive” channel, acknowledging both its sensitivity to mechanical perturbations and the uncertainty regarding the directness of its mechanical gating.

### 2.2 Calcium signal decoding: from transient microdomains to long-term transcriptional reprogramming

Once mechanosensitive ion channels are activated, the most direct downstream effect is Ca^2+^ influx. As one of the most important intracellular second messengers, Ca^2+^ exhibits highly specific spatiotemporal signaling patterns. Soft tissue cells do not respond to mechanical stimuli in a simple “all-or-none” fashion; instead, they finely regulate downstream signaling pathways through the amplitude, frequency, and spatial distribution of Ca^2+^ signals, ultimately shaping their phenotypic fate (Berridge et al., 2003; Prakriya, 2020).

In the spatial dimension, transient Ca^2+^ microdomains are a hallmark of the early phase of mechanosensing. When Piezo1 or TRPV4 opens, Ca^2+^ first rises in a highly localized and short-lived manner in submembrane regions surrounding the channel (Ca^2+^ flickers). These local signals can selectively activate nearby Ca^2+^-dependent effector molecules without substantially changing global cytosolic Ca^2+^ levels, thereby achieving spatially confined signal amplification and branching. Experiments combining traction-force microscopy with endogenous Piezo1 imaging have shown that myosin-II-generated local traction forces can evoke Piezo1-dependent Ca^2+^ flickers at focal adhesions even in the absence of large-scale external loading, and that these events are markedly enriched in stress-concentrated adhesion microdomains (Ellefsen et al., 2019). This indicates that endogenous traction can act as a “microscopic force source” for channel activation, and that Ca^2+^ microdomains function as a spatial filter: on the one hand, ensuring high sensitivity to local mechanical cues, and on the other, avoiding whole-cell Ca^2+^ overload and associated toxicity. While direct imaging of TRPV4-dependent Ca^2+^ flickers in soft tissue settings remains limited, the co-localization of TRPV4 with integrin-based adhesion complexes and its dependence on intact membrane–cytoskeleton coupling suggest that it may similarly generate localized Ca^2+^ microdomains in regions of high cytoskeletal tension (Rahaman et al., 2014; Sharma et al., 2019).

In the temporal dimension, the amplitude, frequency, and duration of mechanical stimuli modulate channel open probabilities and inactivation kinetics, thereby shaping distinct patterns of Ca^2+^ oscillations. Different oscillation patterns are “decoded” by downstream networks into distinct gene expression and functional outputs (Smedler and Uhlén, 2014). As summarized by Smedler and Uhlén, multiple Ca^2+^-dependent transcription factors, notably including NFAT, NF-κB, and CREB, have characteristic response windows to Ca^2+^ oscillation frequency; thus, even under similar total Ca^2+^ loads, cells can adopt different transcriptional programs depending on oscillatory patterns (Berridge et al., 2003; Smedler and Uhlén, 2014). In the context of soft tissue mechanobiology, this implies that low-amplitude, intermittent mechanical stimuli are more likely to induce moderate frequency Ca^2+^ oscillations that favor gene expression associated with tissue homeostasis and modest repair, whereas sustained or high-amplitude mechanical overload tends to trigger high-frequency or near-continuous Ca^2+^ elevation, preferentially activating pro-fibrotic, pro-inflammatory, and cell death pathways (Smedler and Uhlén, 2014; Prakriya, 2020).

Within these “encoding–decoding” networks, YAP/TAZ and NF-κB are two key transcription factors that translate mechanical–Ca^2+^ signals into durable changes in gene expression. YAP/TAZ lie at the crossroads of the Hippo pathway and multiple mechanical signaling routes; their nucleo-cytoplasmic distribution is highly sensitive to ECM stiffness, cytoskeletal tension, and Ca^2+^ levels. It is important to distinguish between two mechanistically distinct routes through which mechanical cues regulate YAP/TAZ.

The first, and more extensively characterized, is the classic “direct mechanical” pathway. ECM stiffness is sensed by integrin-mediated focal adhesions, which transmit force through the talin–vinculin–F-actin molecular clutch to the actomyosin cytoskeleton; Rho/ROCK-dependent actomyosin contractility and stress-fiber formation then promote YAP/TAZ dephosphorylation and nuclear translocation (Dupont et al., 2011; Elosegui-Artola et al., 2017). Force transmission to the nucleus occurs through the LINC (linker of nucleoskeleton and cytoskeleton) complex, which mechanically couples the actin cytoskeleton to the nuclear envelope; sustained tension on the LINC complex alters nuclear pore conformation and facilitates YAP/TAZ nuclear entry independently of changes in Ca^2+^ concentration (Elosegui-Artola et al., 2016). The second route is the Ca^2+^-dependent pathway: in stiffened matrices, Piezo1 activation induces Ca^2+^ influx that triggers p38 MAPK cascades, which can further modulate YAP/TAZ phosphorylation status and promote its nuclear retention (Fu et al., 2021; Li H. et al., 2024).

Current evidence suggests that these two routes operate in parallel and may synergize: the integrin–cytoskeleton–LINC axis establishes the basal set-point of YAP/TAZ nuclear localization according to ECM stiffness, while Piezo1/TRPV4-mediated Ca^2+^ signals provide additional, context-dependent modulation. In this framework, Piezo1 and TRPV4 are best understood as permissive and modulatory rather than strictly necessary or sufficient for YAP/TAZ nuclear translocation in fibrotic settings. Pharmacological inhibition or genetic deletion of Piezo1 attenuates but does not abolish stiffness-induced YAP/TAZ activation in several models (Fu et al., 2021; Rashidi et al., 2025), consistent with the existence of parallel, Ca^2+^-independent mechanical pathways. We therefore use “contributes to” and “facilitates” rather than “drives” when describing the role of Piezo1/TRPV4 in YAP/TAZ regulation throughout this review.

In parallel with its effects on YAP/TAZ, TRPV4 has been identified as a key Ca^2+^ channel that couples mechanical stress to NF-κB activation: in various load and fibrosis models, TRPV4-mediated Ca^2+^ influx activates CaMKII, thereby promoting NF-κB p65 phosphorylation and nuclear translocation and upregulating IL-1β, TNF-α, TGF-β, and other inflammatory and pro-fibrotic mediators. These factors, in turn, enhance TRPV4 expression and activity, forming a positive feedback loop between mechanical cues and inflammation (Zou et al., 2022).

Consequently, in contrast to classic “short-lived tyrosine kinase cascades”, Ca^2+^ signals triggered by mechanosensitive channels more commonly manifest as recurrent, frequency-tunable oscillations or local microdomain events that require multi-layer integration to shape stable fate decisions. Via Ca^2+^-dependent histone-modifying enzymes and chromatin-remodeling complexes, these signals then alter chromatin accessibility at specific loci, effectively “writing” recurrent Ca^2+^ oscillations into stable epigenetic changes (Hernández-Oliveras and Zarain-Herzberg, 2024). Thus, Ca^2+^ microdomains and oscillations induced by mechanosensitive channels are not transient electrophysiological phenomena; rather, through key axes such as Piezo1–p38–YAP/TAZ and TRPV4–CaMKII–NF-κB, they are integrated into transcriptional and epigenetic networks to contribute to the transition of soft tissues from reversible stress responses toward difficult-to-reverse fibrosis and chronic inflammation. In addition, FAK-Src-Ras-ERK cascades can be mechanically activated through integrin clustering without a requisite Ca^2+^ step (Sun et al., 2016), providing a further Ca^2+^-independent route from mechanical force to transcriptional regulation that operates in parallel with the Piezo1/TRPV4–Ca^2+^ axes discussed above.

### 2.3 From biophysics to organism: reconciling in vitro channel properties with in vivo phenotypes

The biophysical properties summarized in Table 2 define the operational range of Piezo1 and TRPV4 under controlled *in vitro* conditions. However, translating these parameters to *in vivo* phenotypes requires consideration of at least three factors that differ systematically between reductionist and organismal settings.

First, the effective mechanical stimulus experienced by a channel *in vivo* is shaped by the ECM spatial filter (Chapter 3). On rigid culture substrates (elastic modulus ∼1 GPa), mechanosensitive channels operate near the upper limit of their activation range; in contrast, the kPa-range stiffness of healthy soft tissues places channels in a substantially different region of their mechanosensitivity curve, where small changes in matrix compliance produce graded rather than saturating responses (M et al., 2025; Verkest et al., 2025). This explains why Piezo1 inhibitors that show robust anti-fibrotic effects on stiff substrates *in vitro* often produce more modest outcomes *in vivo*, because the baseline channel activity being targeted is lower under physiological stiffness conditions.

Second, *in vivo* phenotypes reflect the integrated activity of multiple mechanosensory systems operating in parallel. Genetic deletion of Piezo1 attenuates but does not abolish stiffness-induced YAP/TAZ activation (Fu et al., 2021; Rashidi et al., 2025), consistent with the existence of the Ca^2+^-independent integrin–LINC pathway (Section 2.2). Similarly, TRPV4-null mice are protected from pulmonary fibrosis (Rahaman et al., 2014) but retain largely normal bone mechanoadaptation, indicating that other mechanosensors can functionally compensate in specific tissue contexts. *In vitro* systems, by isolating individual channels, define what a channel can do at its biophysical limit; *in vivo* phenotypes reveal what the channel actually does within a network of parallel pathways, spatial filtering, and homeostatic compensation.

Third, the cellular composition of intact tissues introduces cell-type-specific channel functions that are absent from monoculture assays. For example, TRPV4 in sensory neurons contributes to mechanical allodynia (Mulpuri et al., 2025; Ruviaro et al., 2025), whereas TRPV4 in macrophages regulates phagocytosis and cytokine secretion (Scheraga et al., 2016), and TRPV4 in fibroblasts promotes myofibroblast differentiation (Rahaman et al., 2014). These divergent functions, all triggered by the same channel, converge on a net tissue outcome—fibrosis, pain, or resolution—that cannot be inferred from any single cell-type assay. The gap between *in vitro* channel properties and *in vivo* phenotypes is therefore not a discrepancy to be resolved but a measure of the regulatory complexity of mechanosensory networks, and should be explicitly acknowledged when interpreting loss-of-function or pharmacological intervention studies.

## 3 Physiological spatial filter of soft tissues: depth-dependent mechanical attenuation

**FIGURE 3:**
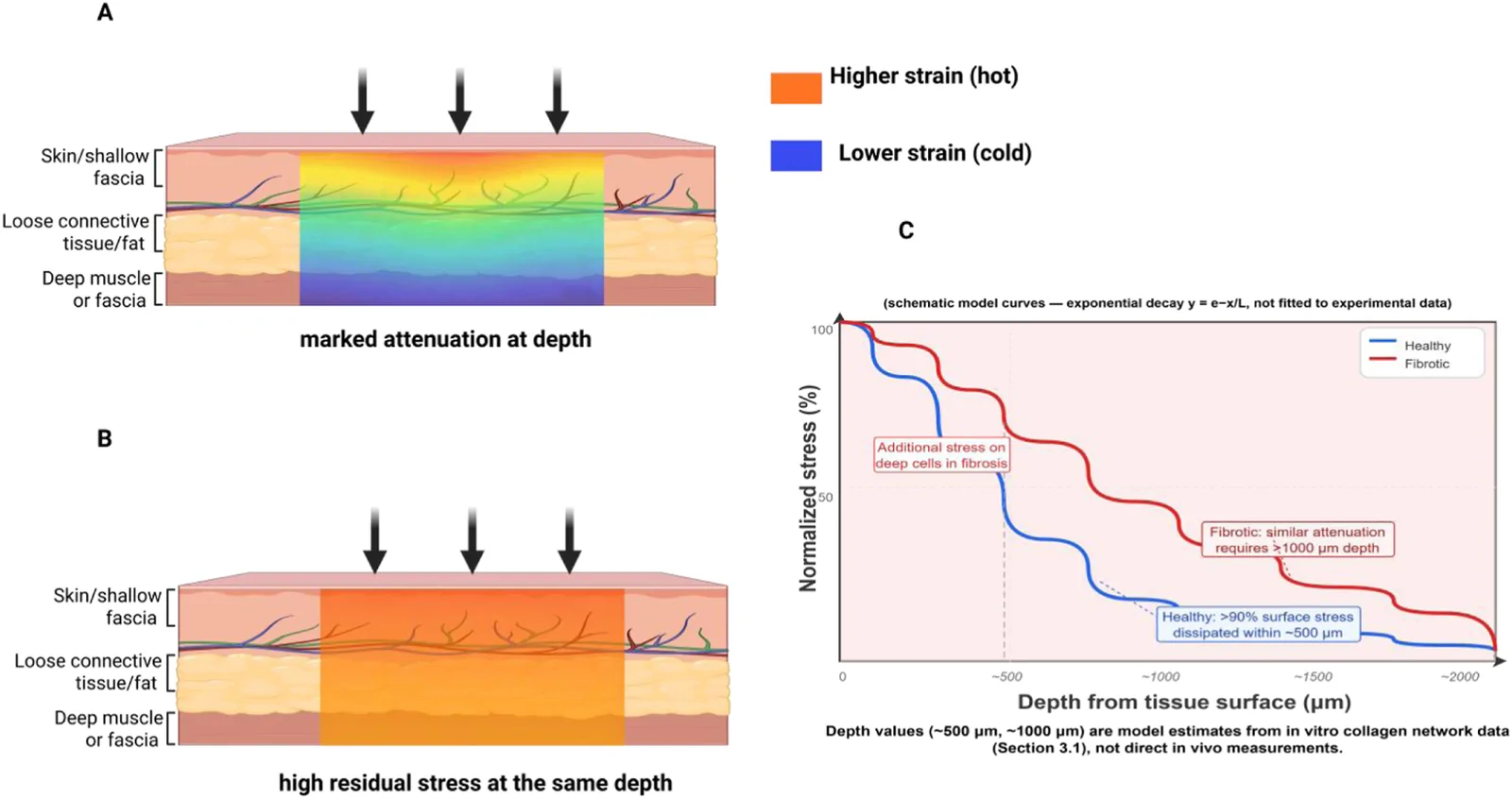
Loss of depth-dependent mechanical attenuation in fibrotic extracellular matrix. **(A)**Under superficial loading (black arrows), normal layered soft tissue shows a depth-dependent strain gradient: high strain (orange/yellow) in skin and shallow fascia, progressively attenuating to low strain (green/blue) in deep muscle or fascia. This marked attenuation at depth protects deep structures from surface loads. **(B)** With similar superficial loading, fibrotic or hardened soft tissue exhibits uniformly high strain (orange) throughout all layers, resulting in high residual stress at the same depth, especially in deep muscle/fascia. Legend:Color scale from orange (higher strain, “hot”) to blue (lower strain, “cold”) schematically represents normalized strain. The loss of depth-dependent attenuation in fibrotic ECM leads to mechanical overload of deep mechanosensitive cells. **(C)** Schematic stress–depth profiles quantifying the spatial filter concept. In healthy tissue (blue curve), normalized stress decays rapidly. In fibrotic tissue (red curve), enhanced cross-linking and reduced viscoelastic dissipation extend the effective attenuation length, exposing deep cells to stress levels that are filtered out in the healthy state. Created in BioRender. w, W. (2026) https://BioRender.com/x6hmi4w.

Mechanical transmission occurs across multiple scales, from the level of the whole organ down to individual cells and even subcellular structures. Understanding mechanical attenuation along this transmission pathway is key to elucidating how mechanosensitive ion channels exert their physiological roles in soft tissues. Current evidence shows that soft tissues are not passive “homogeneous elastic bodies” simply bearing external loads; rather, through the viscoelasticity of the extracellular matrix (ECM), its porous architecture, and non-affine deformation of the fibrous network, they selectively filter mechanical signals in space (Courbot and Elosegui-Artola, 2025). This “spatial filter” property is not merely physical energy dissipation, but a finely tuned biological adaptation: on the one hand, it protects deep cells from excessive mechanical perturbations; on the other, it preserves a specific “usable bandwidth” for local mechanosensing and signal integration (Figure 3).

### 3.1 Porous viscoelasticity and macro–micro strain decoupling

Extensive experimental and theoretical work has shown that tissues and ECM are not ideal linear elastic solids; instead, they display prominent viscoelastic behaviors, including stress relaxation, creep, frequency-dependent stiffness, and marked energy dissipation. This indicates that the tissue response to mechanical loading depends not only on strain or stress amplitude, but also strongly on loading rate, duration, and waveform characteristics (Li Z. Y. et al., 2024; Tan et al., 2025; Voigt et al., 2025).

Within a porous-viscoelastic framework, macroscopic mechanical stimuli applied at the tissue surface typically undergo substantial remodeling in amplitude and temporal profile as they propagate inward. Finite-element simulations and *in vitro* mechanical tests consistently show that ECM fiber networks do not simply undergo affine stretching under load; instead, fiber bending, sliding, rearrangement, and local buckling lead to highly nonlinear and heterogeneous relationships between macroscopic and microscopic strain (Broedersz and MacKintosh, 2014; Shivers et al., 2020; Schütte et al., 2025). Moreover, the nonlinear stiffening of collagen networks has been shown to be controlled by applied stress rather than strain, with network stiffness becoming surprisingly insensitive to collagen concentration, a property that can be quantitatively accounted for by a model of elastic fibers in which normal stresses play a critical role (Licup et al., 2015). In various fiber-network models and tissue-like hydrogels, one can observe large differences in local strain and stress distributions at different depths and topological regions under comparable macroscopic strain, a phenomenon often termed strain/stress localization (Schütte et al., 2025). This is particularly pronounced in collagen-rich ECM, where fiber sliding and rearrangement cause substantial dissipation of external mechanical energy as heat and network restructuring before it reaches the cell membrane (Shivers et al., 2020).

More recently, the mechanisms by which ECM viscoelastic stress relaxation shapes cellular loading have been described quantitatively. Hydrogels with tunable stress-relaxation properties have shown that ECM viscoelasticity provides a time-dependent stress buffer: following an instantaneous load, rapid network rearrangements dissipate internal stress, markedly reducing peak loads experienced by cells at the moment of loading (Chaudhuri et al., 2016). Slowly relaxing matrices allow cells to gradually re-establish mechanical equilibrium under preserved deformation, while fast-relaxing matrices can rapidly reduce cellular stress levels (Li Z. Y. et al., 2024; Tan et al., 2025; Voigt et al., 2025).

To provide a quantitative context, stress relaxation time constants of collagen networks span from sub-second values associated with cell-scale damping to thousands of seconds characteristic of ECM remodeling by fibroblasts (Babaei et al., 2016; Chaudhuri et al., 2016). By comparison, the loading-unloading cycle of human gait has a period of approximately 1 s (frequency ≈1 Hz), which is one to two orders of magnitude shorter than the characteristic relaxation time of many collagen networks. Under healthy conditions, this discrepancy means that during each gait cycle the ECM network has insufficient time to fully transmit surface stresses to deep cells before the load is removed, effectively acting as a low-pass mechanical filter that attenuates high-frequency perturbations. Fibrotic stiffening increases both the elastic modulus and the relaxation time of the ECM, but these two changes have mechanistically distinct consequences: an elevated elastic modulus increases the quasi-static stress amplitude transmitted to cells at any given strain, whereas a prolonged relaxation time means that even low-frequency or sustained loads that were previously buffered by rapid stress relaxation now generate persistent cellular stress (Chaudhuri et al., 2016; Courbot and Elosegui-Artola, 2025; Wu et al., 2025). As a testable prediction, fibrotic ECM cross-linking is expected to extend the effective attenuation length, potentially exposing deep cells to stress levels that would be filtered out under healthy conditions (Broedersz and MacKintosh, 2014; Licup et al., 2015).

From the perspective of “macro–micro strain decoupling,” routine mechanical loads at the tissue surface under physiological conditions (e.g., loads associated with posture maintenance or cyclic stretching during gait) are subject to strong spatial redistribution and temporal remodeling as they traverse multiple ECM layers and interstitial fluid (Chen et al., 2025). For cells residing at depth, this means that even when an organ or tendon as a whole is subjected to sustained loading, the local deformation and stress experienced by deep fibroblasts or immune cells are often far below the macroscopic average and exhibit marked time delays and phase shifts. The combination of nonlinear viscoelastic buffering and non-affine fiber rearrangement thus constitutes a natural spatiotemporal mechanical filter that exposes deep cells to a relatively mild, low-frequency mechanical microenvironment (Courbot and Elosegui-Artola, 2025), thereby establishing a physical basis for the principle that only persistent or significant mechanical abnormalities trigger deep-layer responses (Wu et al., 2025).

It should be acknowledged, however, that the spatial filter concept as applied to intact soft tissues currently rests primarily on *in vitro* hydrogel models and finite-element simulations rather than on direct *in situ* measurements of stress fields at different tissue depths. Techniques such as FRET-based molecular tension sensors, which have been applied to measure piconewton-scale forces across individual adhesion proteins in cultured cells (Grashoff et al., 2010), or intravital strain mapping and embedded micro-scale force transducers, have not yet been systematically deployed to compare stress attenuation in healthy versus fibrotic soft tissues under identical surface loading. The predictions outlined here therefore represent a testable framework that awaits direct experimental validation.

### 3.2 Spatial stratification and threshold calibration of mechanical sensors

In deep soft tissues where there is pronounced strain attenuation and temporal filtering, a natural question arises: how do resident cells such as fibroblasts maintain sufficient mechanosensitivity to very weak, multiply filtered mechanical signals and still mount appropriate responses to long-term matrix changes? Part of the answer lies in the spatial distribution of mechanosensors and the coordinated calibration of their activation thresholds and dynamics.

As introduced in Chapter 2, the spatial distribution of mechanosensors is calibrated to local mechanical demands: Piezo2 predominates in superficial high-strain regions for rapid tactile encoding (Cheng et al., 2026), whereas Piezo1 and TRPV4 are the principal transducers in deep, low-strain compartments where ECM remodeling is the dominant mechanical variable (M et al., 2025; Žavbi et al., 2026).

An additional question concerns how mechanical connectivity is maintained across multiple cell layers within a tissue. At the tissue scale, the integrin–cytoskeleton system does not operate in isolation for individual cells; rather, mechanically coupled cell layers act as a continuous force-transmission chain. Fibroblasts embedded within a dense collagen network are connected to one another through shared ECM fibers and, in some tissues, through direct cell–cell contacts. Mechanical tension applied at the tissue surface is transmitted through this layered architecture: superficial cells, via their integrin-based adhesions, pull on ECM fibers that, in turn, transmit force to the adhesions of deeper cells, creating a series of cell–matrix–cell mechanical relays (Elosegui-Artola et al., 2016; Sun et al., 2016). This layered force chain is thought to experience substantially less attenuation than force transmitted through the lipid bilayer alone, because collagen fibers and the cytoskeletal networks they connect to are orders of magnitude stiffer than the plasma membrane and do not undergo the same degree of viscous dissipation. Consequently, even when superficial membrane physical properties remain relatively constant, the integrin–cytoskeleton–ECM chain can function as a sensitive detector of changes in ECM stiffness and cross-linking at depth (Chanduri et al., 2024; Jaddivada et al., 2026).

This distinction between the lipid bilayer route and the integrin–cytoskeleton route also helps to address a related mechanistic question: why, upon ECM stiffening, the integrin–cytoskeleton system registers substantial changes in mechanical load while the physical properties of the plasma membrane outside adhesion sites may remain largely unchanged. The answer likely lies in the differential mechanical properties of the two force-transmission routes. The lipid bilayer has a bending stiffness on the order of ∼10–20 k_BT and can readily accommodate moderate tension changes through shape fluctuations and membrane reservoir recruitment without a proportional change in lateral tension sensed by dispersed membrane proteins (Wiggins and Phillips, 2004). In contrast, talin, the core mechanosensitive element of the molecular clutch, contains multiple helical bundle domains in its rod region that unfold at defined force thresholds. Mechanical tension of approximately 5 pN is sufficient to trigger the unfolding of individual talin rod domains, exposing cryptic vinculin-binding sites (VBSs) in a switch-like manner. Once exposed, these VBSs recruit vinculin, which in turn binds F-actin, creating a ratchet-like mechanism that locks talin in an extended conformation and amplifies small increases in ECM stiffness into sustained changes in cytoskeletal tension (Goult et al., 2018; Rangarajan et al., 2025).

Viewed from a spatial-stratification perspective, the mechanosensory apparatus of soft tissues can thus be summarized as follows: superficial high-strain regions rely primarily on low-threshold, fast-kinetic channels such as Piezo2 for “front-line high-frequency detection” (Cheng et al., 2026), whereas deep low-strain regions use relatively higher-threshold, slower channels such as Piezo1 and TRPV4 to sense ECM stiffness changes and accumulated traction over long time scales (M et al., 2025; Žavbi et al., 2026). This “spatial–threshold co-calibration” allows cells at different depths to take on distinct roles in mechanical information processing: the former are specialized for detecting brief acute stimuli and generating rapid neural/sensory outputs; the latter are biased toward chronic mechanical remodeling and matrix metabolic regulation.

It is worth noting that, although the functional coupling between Piezo1 and FAs has been consistently demonstrated by imaging and functional experiments, the precise atomic-level binding sites between intracellular domains of Piezo1 and specific cytoskeletal tethers (such as talin, vinculin, and α-actinin) remain undetermined (Verkest et al., 2025). Recent cryo-EM structures of human Piezo1 have identified several intracellular structural elements that are potential interaction interfaces—including a long beam helix supporting the transmembrane domain, disordered N-terminal THU1/2 regions, and a cytosolic plug domain, but direct atomic-level visualization of Piezo1 bound to talin or vinculin has not yet been achieved (Shan et al., 2025). This represents an important knowledge gap at the interface of structural biology and mechanobiology.

### 3.3 Failure of the spatial filter in pathological states and malignant positive feedback

In healthy tissues, ECM viscoelasticity, non-affine fiber deformation, and porous architecture jointly maintain the function of the mechanical spatial filter: on the one hand, they buffer and attenuate loads so that deep cells are not exposed to excessively high mechanical stress; on the other hand, they still allow essential mechanical signals to be integrated into long-term homeostatic regulation (Courbot and Elosegui-Artola, 2025; Wu et al., 2025). Under conditions of chronic injury, inflammation, or metabolic disturbance, however, ECM composition and structure undergo profound remodeling, and we propose that this physiological filter may be among the first systems to become compromised.

In multiple organ fibrosis models, pathological collagen over-deposition and enhanced cross-linking (e.g., via lysyl oxidase) are suggested to convert the ECM from a viscoelastic network toward a more rigid, brittle state (Ganzleben and Medoff, 2025; Lin et al., 2025). In parallel, ECM stress-relaxation and energy-dissipation capacities decline markedly: under similar macroscopic strain, stiffened tissues are predicted to respond with higher internal stresses and shorter delays (Courbot and Elosegui-Artola, 2025; Lin et al., 2025; Wu et al., 2025). From a mechanical-transmission standpoint, this means that mechanical energy previously dissipated through fiber rearrangement and matrix flow is now delivered more directly, as a “rigid signal,” to the cell–ECM interface and cytoskeleton, raising both the peak magnitude and duration of stresses experienced by deep cells (Chang et al., 2025; Chen et al., 2026).

Within such a stiffened microenvironment, the “operating point” of mechanosensitive ion channels is hypothesized to be systematically shifted upward. Recent reviews and primary studies have shown that increased ECM stiffness significantly raises Piezo1 activation probability, making it more readily opened under a given level of endogenous traction (M et al., 2025; Verkest et al., 2025). In fibrotic models of kidney, lung, liver, and myocardium, excessive Piezo1 activation upregulates downstream effectors such as p38 MAPK and YAP/TAZ through Ca^2+^-dependent pathways, contributing to an α-SMA^+^ myofibroblast phenotype and promoting massive secretion of collagen and other ECM components (Chang et al., 2025; Lin et al., 2025).

TRPV4 exhibits a similarly “hypersensitized” response in multi-organ fibrosis and chronic inflammation after filter compromise. ECM stiffening often co-occurs with increased activity of TGF-β family factors, which not only upregulate TRPV4 expression but also modify its gating properties in ways that make it more easily activated by a given mechanical input (Rahaman et al., 2014; Žavbi et al., 2026). TRPV4-mediated Ca^2+^ influx, acting through CaMKII, RhoA/ROCK, and other downstream kinase cascades, enhances cellular contractility, promotes stress-fiber formation, and increases ECM synthetic capacity (Rahaman et al., 2014; Voigt et al., 2025; Žavbi et al., 2026). In airway inflammation models, abnormal mechanical microenvironments interact with nociceptors to further amplify neurogenic inflammation (Wang et al., 2026). Overall, TRPV4 contributes to mechano-inflammatory-fibrotic networks in the lung, heart, and other organs through cross-talk with NF-κB and YAP/TAZ pathways (Zou et al., 2022; Žavbi et al., 2026).

**FIGURE 4:**
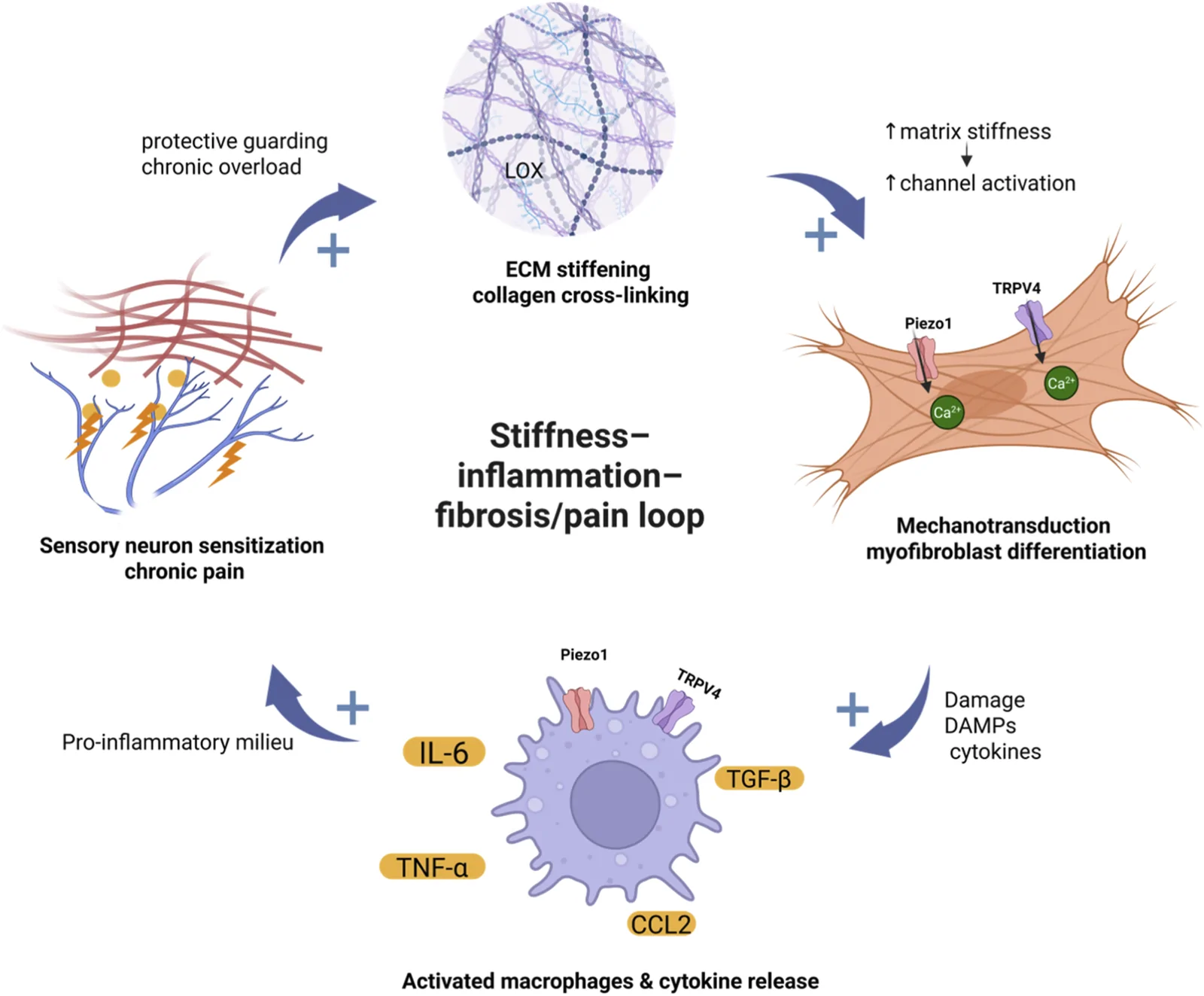
Multicellular positive feedback loop linking ECM stiffening, inflammation, fibrosis and pain (top). LOX-mediated collagen cross-linking increases matrix stiffness, reducing normal mechanical dissipation and predisposing to mechanosensitive channel overactivation (right). Fibroblasts sense the stiffened ECM via Piezo1/TRPV4-mediated Ca^2+^ influx, differentiate into myofibroblasts with α-SMA stress fibers and overproduce ECM, further enhancing stiffness and damage-associated signals (DAMPs, cytokines) (bottom). Macrophages in this environment are activated and secrete IL-6, TNF-α, TGF-β, CCL2 and other mediators, creating a pro-inflammatory milieu that amplifies fibroblast activation and ECM remodeling (left). Persistent mechanical overload and inflammatory mediators sensitize peripheral sensory endings (e.g., via Piezo2/TRPV4), leading to chronic pain, protective guarding and altered use. These maladaptive loading patterns in turn aggravate ECM stiffening. Clockwise arrows with “+” signs indicate a self-reinforcing multicellular positive feedback loop connecting stiffness, inflammation, fibrosis and pain. Created in BioRender. w, W. (2026) https://BioRender.com/xxbkigt.

Taken together, pathological ECM stiffening is proposed to lead to progressive spatial filter compromise, which may ultimately establish a malignant positive-feedback loop that is difficult to reverse. Once ECM stiffness exceeds a certain threshold, viscoelastic dissipation is markedly reduced and macroscopic loads are transmitted more directly to deep cells, raising the activation frequency of Piezo1 and TRPV4 and sustaining elevated activity in Ca^2+^-dependent fibrotic and inflammatory pathways. These over-activated pathways in turn drive further ECM deposition and cross-linking, increasing stiffness and further reducing stress-relaxation capacity, a cycle that helps explain why many chronic soft-tissue disorders become difficult to reverse with single anti-inflammatory or anti-fibrotic agents once a certain stiffness threshold has been exceeded (Chang et al., 2025; Gromakovskis, 2025; Chen et al., 2026). Within this framework, combining “restoration of ECM viscoelasticity and spatial-filter function” with “precise modulation of Piezo1/TRPV4 activity” provides a mechanobiologically grounded rationale for future combined mechanical-biological interventions (Figure 4).

We emphasize that while the evidence from *in vitro* hydrogel models and computational simulations reviewed above provides a mechanistically plausible framework for filter failure, direct experimental confirmation-using the *in situ* techniques discussed in Section 3.1—that deep cells in fibrotic tissues experience higher stress under identical surface loading compared with healthy tissues remains an important goal for future studies.

## 4 Structural collapse and hardening: Piezo/TRPV4-driven microenvironmental distortion

As soft tissues transition from physiological homeostasis to pathological states, mechanosensitive ion channels are no longer merely passive “sensors” of external loads; rather, they gradually become “effectors” that may progressively promote structural remodeling of the tissue. Through multilayered signaling cascades, channels such as Piezo1 and TRPV4 translate aberrant mechanical inputs into persistent extracellular matrix (ECM) deposition and progressive tissue stiffening. This process involves the cytoskeleton–molecular clutch system, fibroblast phenotypic transformation, and coordinated regulation of the immune microenvironment, constituting a key mechanobiological basis for soft tissue fibrosis and chronic hardening lesions (Dong L. et al., 2025; Ganzleben and Medoff, 2025; Lei et al., 2025; Lin et al., 2025).

### 4.1 The vanguard of mechanical sensing: integrin–molecular clutch coupling with Piezo1

**FIGURE 5:**
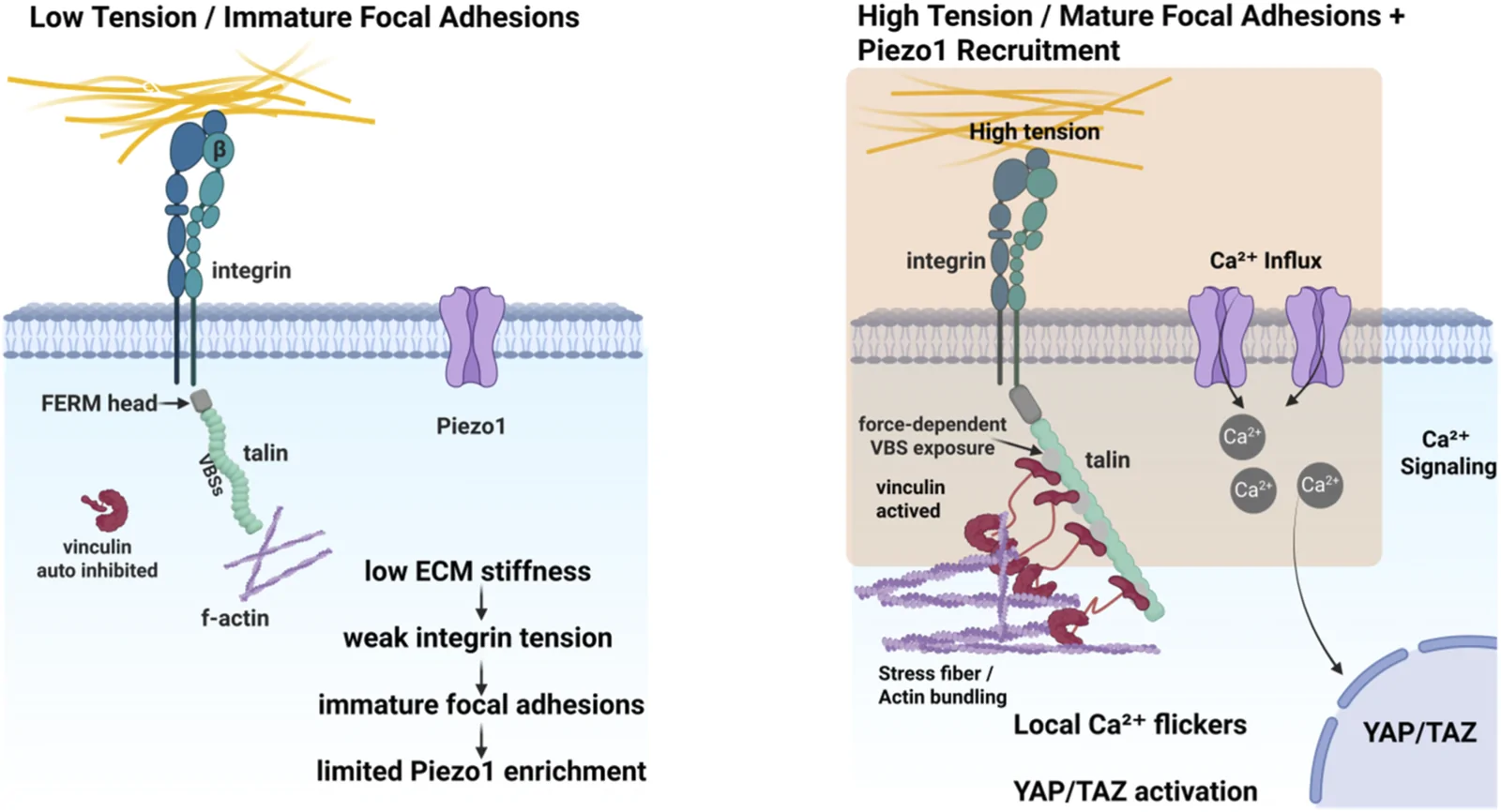
Force-dependent recruitment of Piezo1 to integrin-based focal adhesions Left: Low tension/immature focal adhesions. ** On compliant ECM (low ECM stiffness), weak integrin tension leaves focal adhesions immature. Under this low-tension condition, the talin rod domain remains folded with its vinculin-binding sites (VBSs) masked, vinculin remains autoinhibited, and F-actin filaments are discontinuous. Piezo1 channels remain diffusely distributed along the plasma membrane, with limited Piezo1 enrichment at these immature adhesion sites. Right: High tension/mature focal adhesions + Piezo1 recruitment. On stiff ECM, high integrin tension drives the force-dependent unfolding of the talin rod domain, exposing VBSs to recruit and activate multiple vinculin molecules. Activated vinculin bridges talin to F-actin, promoting stress fiber formation and actin bundling to mature the focal adhesions. Consequently, Piezo1 channels cluster at these high-tension adhesion sites, where mechanical force drives Ca^2+^ influx and local Ca^2+^flickers, which downstream activate YAP/TAZ signaling. The schematic highlights how ECM stiffness and integrin–talin–vinculin “molecular clutch” mechanics regulate Piezo1 spatial distribution and mechanosensitive Ca^2+^ signaling. Created in BioRender. w, W. (2026) https://BioRender.com/23yi4en.

As discussed in Chapters 2 and 3, integrin-mediated focal adhesions (FAs) and the molecular clutch, formed by integrins, talin, vinculin, and F-actin, serve as the core bridge through which mechanical signals are transmitted from the ECM to the cytoskeleton (Elosegui-Artola et al., 2016; Sun et al., 2016). Under physiological conditions, moderate tension enables reversible engagement and load sharing among clutch components; when the ECM becomes pathologically stiffened, sustained high tensile force drives talin domain unfolding, increased vinculin recruitment, and FA stabilization, converting the clutch into a high-load state that supports long-lasting high-tension microdomains at FAs(Wang et al., 2021; Chanduri et al., 2024; Wang H. et al., 2024). Against this background, Piezo1 is force-dependently recruited to mature FAs, where it generates local Ca^2+^ influx events that contribute to adhesion growth and remodeling (Yao et al., 2022). Taken together with findings that Piezo1-dependent Ca^2+^ flickers are enriched in regions of high traction force (Ellefsen et al., 2019), these results indicate that under ECM stiffening conditions, the molecular clutch creates a stable high-tension microenvironment at FAs that provides an important mechanical prerequisite for directional recruitment and mechanical activation of Piezo1 (Figure 5).

Although TRPV4 is not a canonical structural component of FAs, substantial evidence indicates that its mechano-responsive gating also depends on the integrin–cytoskeleton system and that it participates in matrix stiffening during endothelial-to-mesenchymal transition (EndMT), valvular interstitial cell activation, and foreign-body responses (Rahaman et al., 2014; Dutta et al., 2025; Mukherjee et al., 2025). In many cell types, TRPV4 activation coincides with RhoA/ROCK-mediated stress-fiber formation and traction-force enhancement; the upstream mechanical signal may be membrane tension *per se* or tension transmitted through the integrin–cytoskeleton network (Rahaman et al., 2014; Dutta et al., 2025; Mukherjee et al., 2025). From a systems perspective, Piezo1 and TRPV4 share a common “front end” consisting of integrin, talin/vinculin, and F-actin within the molecular clutch. Although they assume different functions and operate on different time scales across cell subsets and organs, together they constitute the vanguard mechanosensory apparatus in stiffened microenvironments.

While Piezo1 is force-dependently recruited to integrin-based focal adhesions, TRPV4 exhibits a broader subcellular distribution. Beyond its functional coupling to β1-integrin at cell-matrix adhesions (Matthews et al., 2010; Sharma et al., 2019), TRPV4 is enriched at cell-cell junctions in epithelial barriers, as demonstrated in keratinocytes, where it forms complexes with β-catenin at adherens junctions and contributes to barrier function (Sokabe et al., 2010). This multi-site distribution—focal adhesions and adherens junctions—may explain TRPV4’s broader and more context-dependent functional roles compared with Piezo1 in soft tissue mechanobiology.

### 4.2 Fibroblast activation and the “force–fibrosis” positive feedback

Fibroblasts are the most abundant resident stromal cells in soft tissues and are the primary executors of ECM synthesis and turnover. Under physiological conditions, fibroblasts remain in a low-synthesis, low-contractility steady state, thereby maintaining slow ECM renewal and microenvironmental homeostasis. Once subjected to sustained mechanical or inflammatory stimuli, they differentiate into myofibroblasts with high contractility and elevated ECM synthetic capacity, which is the central cellular event underlying fibrosis (Ganzleben and Medoff, 2025; Lin et al., 2025).

Multiple studies suggest that Piezo1 is highly expressed in fibroblasts and myofibroblasts across various organs and plays an important role in sensing ECM stiffness and cyclic stretch (Dong L. et al., 2025; Lin et al., 2025). In skin fibrosis models, increased matrix stiffness not only upregulates Piezo1 expression but also activates downstream inflammatory and fibrotic pathways (such as the Wnt2/Wnt11–CCL24 axis) via Piezo1-mediated Ca^2+^ signaling, promoting fibroblasts to secrete more collagen and chemokines and thereby forming a “stiffness–Piezo1–inflammation/fibrosis” positive feedback loop (He et al., 2024). In organs such as the lung, kidney, and liver, Piezo1 has likewise been shown to participate in mechanical activation of myofibroblasts and massive ECM deposition; inhibiting Piezo1 activity can alleviate fibrosis to varying degrees in multiple animal models (Dong L. et al., 2025; Lin et al., 2025).

At the level of signal transduction, excessive Piezo1 activation typically triggers Ca^2+^ influx and thereby activates kinase networks including CaMKII, p38 MAPK, and FAK/Src, forming functional coupling with the mechanosensitive transcriptional coactivator YAP/TAZ. The nuclear translocation of YAP/TAZ is highly sensitive to ECM stiffness, cytoskeletal tension, and Ca^2+^ signals; in many fibrosis models, it has been suggested to be a central signaling hub that maintains the myofibroblast phenotype and contributes to excessive ECM deposition (Liu et al., 2015; Papavassiliou et al., 2024; Rashidi et al., 2025).

TRPV4 plays a particularly notable role in fibroblast activation through synergy with the TGF-β pathway. Existing studies show that TGF-β can upregulate TRPV4 expression and promote its insertion into the plasma membrane, while Ca^2+^ signals generated upon TRPV4 activation further enhance the activity of TGF-β/Smad and PI3K–AKT pathways, promoting myofibroblast formation and ECM stiffening in multiple organs such as the heart and lung (Rahaman et al., 2014; Sharma et al., 2019). More recent work indicates that in stiffened matrix environments, TRPV4-mediated Ca^2+^ events promote stress-fiber formation, traction-force enhancement, and the assembly of collagen–fibronectin complexes, thereby amplifying fibrotic progression at both the mechanical and synthetic levels (Rahaman et al., 2014).

Taken together, current evidence allows the “force–fibrosis” positive feedback to be summarized as follows: increased ECM stiffness leads to prolonged loading of the integrin–molecular clutch, resulting in increased activation frequency and open duration of Piezo1/TRPV4 in high-tension microdomains; this in turn induces persistent activation of Ca^2+^-dependent YAP/TAZ, Smad, and stress-kinase pathways; fibroblasts consequently differentiate into α-SMA^+^ myofibroblasts and markedly increase synthesis of collagens and ECM cross-linking enzymes; ECM thereby becomes further stiffened and its viscoelastic dissipation capacity is reduced; the “operating point” of mechanosensitive channels shifts upward and their effective activation thresholds decrease, manifesting over time as graded amplification of positive feedback.

It should be noted, however, that this process is currently supported mainly by evidence from individual models and indirect observations, and systematic, causally definitive validation across multiple organs and models is still lacking.

### 4.3 Immune microenvironment crosstalk amplifying the hardening process

Soft tissue fibrosis does not arise from the abnormal behavior of a single cell type, but rather from the interplay of multiple cell populations, among which macrophages serve as key amplifiers in the interconnected processes of stiffness, inflammation, and fibrosis (Lei et al., 2025). In recent years, Piezo1 and TRPV4 have increasingly been recognized as important mechanosensitive channels in immune cells, particularly regarding their roles in macrophage sensing of matrix stiffness, shear stress, and exogenous foreign bodies (Wang Y. et al., 2024; Liu et al., 2025).

In macrophages, Piezo1 has been shown to be an important mechanoreceptor for ECM stiffness and blood-flow shear stress, with its activation able to regulate polarization states and cytokine secretion profiles through Ca^2+^-dependent mechanisms (Luo T. et al., 2023; Wang Y. et al., 2024). In fibrosis and inflammation models of organs such as the liver and lung, Piezo1 activation can induce or maintain pro-inflammatory/pro-fibrotic phenotypes, upregulating IL-6, TNF-α, CCL2, and other factors, and amplifying inflammation and fibroblast activation via Notch, NF-κB, or STAT pathways (Luo T. et al., 2023). Conversely, in some models, enhanced Piezo1-mediated stiffness sensing has been found to facilitate macrophage phagocytosis and clearance of apoptotic cells (efferocytosis), thereby promoting the resolution of inflammation and fibrosis (Wang Y. et al., 2024), highlighting the marked context-dependence of Piezo1 function within the immune microenvironment.

TRPV4 also exhibits bidirectional regulatory features in macrophages and other immune cells. On the one hand, by sensing matrix stiffening and mechanical stretch, TRPV4 can promote TGF-β activation and myofibroblast differentiation and thus plays a pro-fibrotic role in lung fibrosis and other organ pathologies (Rahaman et al., 2014). On the other hand, in certain acute inflammation and foreign-body reaction models, TRPV4 also participates in limiting excessive inflammation or modulating phagocytic behavior (Scheraga et al., 2016). Overall, TRPV4 tends to act as a Ca^2+^ entry point within the “mechanical–inflammation–fibrosis” axis, with its specific effects depending on the microenvironment, cell type, and coexisting signals (such as TGF-β and IL-1β), dynamically balancing between pro-inflammatory/pro-fibrotic and anti-inflammatory/regulatory states.

Integrating the above evidence, the fibrotic process can be abstracted into a cross-cell-type mechanobiological–immune crosstalk pathway: ECM hardening and stress concentration first activate Piezo1/TRPV4 in fibroblasts, leading to myofibroblast formation and excessive ECM deposition and thereby further disturbing the local mechanical microenvironment; subsequently, resident and infiltrating macrophages sense stiffness and fluid shear changes through the Piezo1/TRPV4 axis, polarize toward pro-inflammatory/pro-fibrotic lineages, and secrete TGF-β, IL-6, TNF-α, CCL2, and other factors; these mediators, via paracrine signaling, activate or stabilize fibroblast/myofibroblast phenotypes with high ECM synthetic capacity. At the tissue scale, stiffness, inflammation, and fibrosis thus form a closed, mutually reinforcing loop that substantially increases the difficulty of disease reversal (Wang Y. et al., 2024).

## 5 Neural sensitization and pain: mechanical–chemical dual overload in a hardened microenvironment

Traditional views hold that chronic soft tissue pain is mainly driven by persistent inflammation, peripheral nerve injury, and central sensitization. More recent research has progressively recognized that tissue stiffening itself can also act as an important direct contributor of chronic pain (Wan et al., 2024; Lin et al., 2025). Abnormal changes in matrix mechanical properties, on one hand, directly activate sensory nerve endings through local mechanical stress concentration; on the other hand, they lower the activation thresholds of mechanosensitive channels via inflammatory mediators, thereby inducing and maintaining peripheral sensitization. In this process, Piezo2 and TRPV4 channels form important molecular hubs that translate “hardness–stress” into “pain–inflammation” signals (Sánchez-Carranza et al., 2024; Wan et al., 2024; Gromakovskis, 2025; Ruviaro et al., 2025).

In fibrotic or hardened soft tissues, the ECM undergoes marked remodeling: collagen fiber deposition increases, pathological cross-linking is enhanced, and the distribution of free water and glycosaminoglycans becomes imbalanced, resulting in increased overall tissue stiffness and decreased viscoelastic dissipation capacity (Lin et al., 2025; Chen et al., 2026). More importantly, this remodeling is highly spatially heterogeneous. Tensile or compressive loads within the tissue are not uniformly distributed, but instead tend to form local “stress hotspots” in regions of structural discontinuity or abnormal fiber orientation (Gehwolf et al., 2025; Plaut, 2025; Chen et al., 2026). Fascial networks and tendon entheses are typical examples: after chronic overload or impaired healing, high-stiffness cords can form in fascia and scar regions, where the surrounding ECM exerts strong mechanical traction on densely distributed sensory nerve endings while simultaneously accumulating multiple inflammatory and sensitizing mediators (Gromakovskis, 2025).

Free endings of primary sensory neurons express high levels of Piezo2 and several transient receptor potential (TRP) channels, including TRPV4. Piezo2 is widely considered a key transducer of mechanical pain and touch, whereas TRPV4 participates in the regulation of multiple forms of mechanical and inflammatory pain (Wan et al., 2024; Ruviaro et al., 2025; Xie et al., 2026). Existing animal model studies have shown that in the context of inflammatory arthritis, osteoarthritis, and neuropathic pain, Piezo2 is upregulated in a substantial fraction of nociceptive fibers, and its genetic deletion or pharmacological blockade significantly alleviates mechanical hyperalgesia and mechanical allodynia (Sánchez-Carranza et al., 2024; Wan et al., 2024). Inhibition of Piezo2 mechanical gating using small molecules or dyes also attenuates mechanically evoked pain in mice (Sánchez-Carranza et al., 2024). With respect to TRPV4, multiple models, including cancer pain, diabetic neuropathy, and complex regional pain syndrome, demonstrate a close association between TRPV4 activity and mechanical allodynia: TRPV4 antagonists dose-dependently mitigate reductions in mechanical pain threshold, while having relatively modest effects on heat pain sensitivity (Mulpuri et al., 2025; Ruviaro et al., 2025). At the spinal level, TRPV4 expressed in microglia and along glial–neuronal axes has been shown to contribute to the amplification of central mechanical pain hypersensitivity following peripheral nerve injury (Ruviaro et al., 2025). Thus, under conditions of ECM stiffening and impaired spatial filtering, Piezo2/TRPV4^+^ endings located in high-tension microregions are repeatedly exposed to abnormal mechanical stresses even during daily loading or minor activities, giving rise to frequent, overly large depolarization events that manifest clinically as primary mechanical hyperalgesia.

In parallel with the physical stress pathway, the hardened matrix continuously reshapes the local chemical milieu through the immune–inflammatory axis, deepening “chemical sensitization” of mechanosensitive channels. As discussed in Chapter 4, macrophages sense ECM stiffening through Piezo1 and TRPV4 and, depending on the context, polarize toward pro-inflammatory/pro-fibrotic phenotypes or promote resolution of inflammation via efferocytosis (Scheraga et al., 2016; Luo S. et al., 2023; Wang Y. et al., 2024). Prolonged high-tension contraction of myofibroblasts and abnormal ECM deposition further sustain the release of damage-associated molecular patterns (DAMPs) and pro-inflammatory cytokines (Scheraga et al., 2016).

On this “stiffness–inflammation” biochemical backdrop, large quantities of inflammatory mediators, on one hand, promote the shift of fibroblasts toward α-SMA^+^ myofibroblast phenotypes and amplify ECM synthesis and cross-linking; on the other hand, they directly or indirectly sensitize mechanosensitive channels on sensory neurons via multiple G protein-coupled receptors (such as EP, BK, and PAR2) and downstream PKA/PKC cascades (Mulpuri et al., 2025). TRPV4 channel activity and membrane trafficking are finely tuned by phosphorylation through kinases such as PKA, PKC, Src, and SGK1, and certain inflammatory mediator–receptor axes (for example, PAR2–PLCβ–PKA/PKC) can markedly enhance TRPV4 sensitivity to mechanical, osmotic, and chemical stimuli. TRPV4 deletion or blockade significantly reduces mechanical hyperalgesia in corresponding models (Mulpuri et al., 2025). For Piezo2, recent systematic studies of mechanical pain and evoked pain have shown that in inflammatory or neuropathic contexts, Piezo2 not only becomes upregulated but also exhibits altered mechanical response thresholds and kinetic properties; inhibiting Piezo2 activity can significantly alleviate mechanical hypersensitivity in multiple models (Sánchez-Carranza et al., 2024; Habib et al., 2025).

Taken together, ECM hardening, immune activation, and channel sensitization form a mutually reinforcing network. Consequently, restoring ECM viscoelasticity, modulating Piezo/TRPV activity, and reestablishing mechanical homeostasis are important strategies for breaking this loop and reversing chronic soft tissue pain.

## 6 Breaking the malignant closed loop: targeted interventions and the mechanical therapeutic window

**FIGURE 6:**
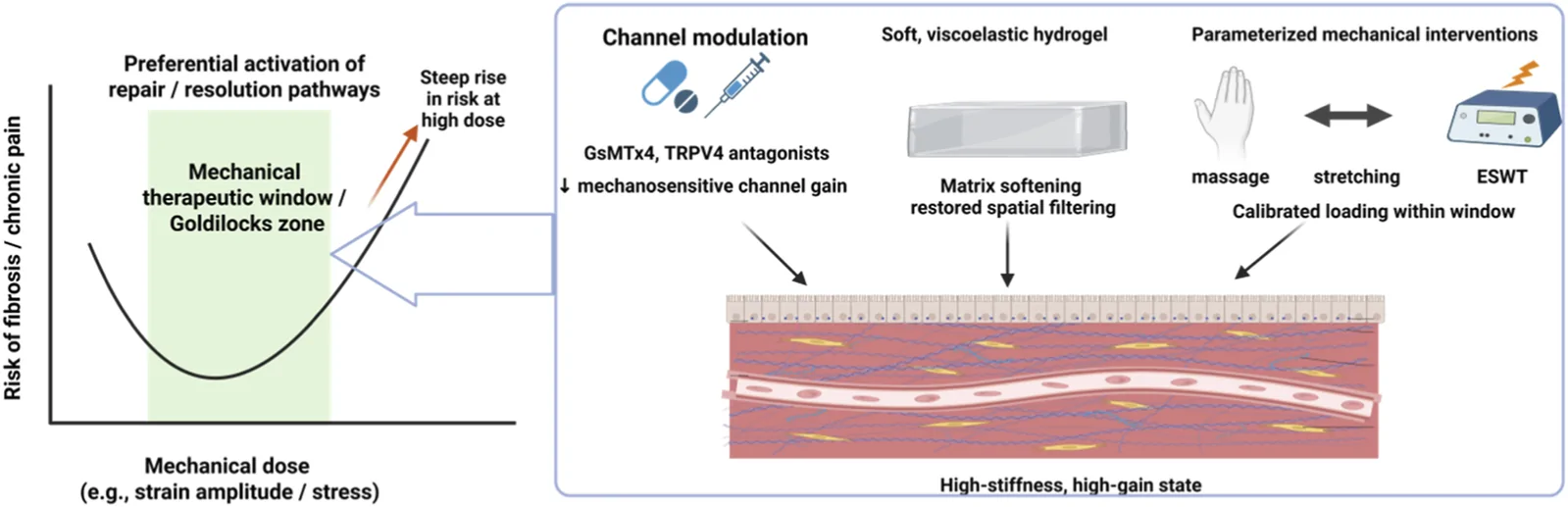
Mechanically defined therapeutic window and interventions targeting Piezo/TRPV4–ECM networks. Left: Mechanical simulated dose–response curve. Risk of fibrosis/chronic pain (y-axis) is plotted against mechanical dose (x-axis; e.g., strain amplitude/stress). A U-shaped curve indicates increased risk at both very low and very high doses, with an intermediate Mechanical therapeutic window/Goldilocks zone (green band) where preferential activation of repair/resolution pathways is expected. At high doses, risk rises steeply (orange arrow). Right: Multimodal interventions shifting a high-stiffness, high-gain state back into the therapeutic window. A pathological “high-stiffness, high-gain state” of the ECM–cell system is illustrated at the bottom. Three classes of interventions act in a different manner: Channel modulation. Agents such as GsMTx4 and TRPV4 antagonists reduce mechanosensitive channel gain. Soft, viscoelastic hydrogels. ECM-mimetic, compliant materials soften the effective matrix and restore spatial filtering of mechanical stress. Parameterized mechanical interventions. Calibrated manual therapy, stretching and ESWT deliver mechanical loading within the therapeutic window. A large blue hollow arrow from the intervention panel toward the green zone emphasizes that combined strategies seek to move the system from a high-stiffness, high-gain regime back into a mechanically defined therapeutic window. Created in BioRender. w, W. (2026) https://BioRender.com/uwottdk.

Given that the preceding sections have characterized chronic soft tissue pathology as a “stiffness–inflammation–pain” malignant closed loop formed by ECM hardening, inflammatory amplification, and neural sensitization, the rational aim of intervention strategies should not be merely to suppress a single pathway. Instead, they should seek to “reduce loop gain” at multiple levels and restore mechanobiological homeostasis of the tissue. This chapter discusses representative current approaches and potential translational pathways from three angles: pharmacology and materials science, non-invasive mechanical intervention, and the “mechanical therapeutic window” (Figure 6).

### 6.1 Pharmacology and materials science: from channel inhibition to matrix remodeling

At the molecular level, directly targeting mechanosensitive channels is one of the most straightforward strategies. GsMTx4, a peptide derived from tarantula venom, is among the most extensively studied inhibitors of mechanosensitive channels. Its mode of action does not involve specific binding to channel proteins such as Piezo1; rather, it inserts into the outer leaflet of the lipid bilayer, altering local membrane tension and curvature and thereby increasing the energy threshold for opening mechanically gated channels (Gnanasambandam et al., 2017). Animal experiments show that in fibrosis models of organs such as the kidney and uterus, appropriate dosing of GsMTx4 attenuates Piezo1-mediated Ca^2+^ influx and its downstream pro-fibrotic and pro-inflammatory signaling, reduces pathological ECM deposition, and improves tissue structure and function (Bae et al., 2011; Lei et al., 2026). This suggests that, within this positive-feedback cycle, mechanical modulation at the “membrane–channel” interface can effectively dampen pathological amplification by mechanosensitive channels.

The development of small-molecule antagonists targeting TRPV4 represent another important pharmacological direction. Highly selective TRPV4 blockers have been shown in multiple animal models to suppress Ca^2+^ influx induced by mechanical or osmotic stress and to reduce edema, vascular leakage, mechanical hyperalgesia, and tissue fibrosis (Cheung et al., 2017; Dong Q. et al., 2025). Recent pharmacological studies indicate that TRPV4 inhibition exhibits potential to mitigate Ca^2+^ overload and tissue stiffening in ischemia–reperfusion injury and fibrosis of organs such as the heart, lung, and kidney (Dong Q. et al., 2025). However, because TRPV4 is broadly involved in osmotic regulation, alveolar barrier maintenance, and immune cell function, long-term systemic antagonism may entail non-trivial side effects. Thus, more realistic directions include local administration, short-course interventions, or small molecules with biased regulatory properties that transiently downregulate TRPV4 activity at lesion sites.

Complementing the idea of “turning off channels” with small molecules, materials science offers a strategy of “resetting the microenvironment set point.” In recent years, soft matrix hydrogels that mimic the mechanical properties of healthy soft tissues have been widely used in fibrosis-reversal studies. Viscoelastic hydrogels can, through “tension shielding” and time-dependent stress relaxation, physically share and redistribute local traction forces, thereby reducing the actual stress borne by fibroblasts and endothelial cells from a “pathological hardening range” down toward physiological levels (Adapala et al., 2021; Zhang et al., 2026). In lung, intervertebral disc, and myocardial models, such hydrogels have been shown to downregulate genes associated with myofibroblast/collagen phenotypes (e.g., α-SMA, COL1A1), inhibit YAP/TAZ nuclear translocation and mechanosensitive pathways, promote matrix metalloproteinase expression and ECM remodeling, and thereby achieve decreased stiffness and “re-softening” of the microenvironment (Yu et al., 2025; Zhang et al., 2026). When these hydrogels are further loaded with antifibrotic small molecules, siRNA, immune modulators, or engineered CAR-macrophages, they exhibit more durable and spatially targeted anti-hardening effects in lung, cardiac, and subcutaneous fibrosis models than single-agent drug regimens (Yu et al., 2025; Lu et al., 2026).

Thus, from the perspective of closed-loop control, small-molecule inhibitors mainly act by lowering channel “amplification gain,” whereas soft matrices and viscoelastic materials work by “resetting boundary conditions” to pull the mechanical operating point of the entire network back into a homeostatic range. The two approaches are highly complementary in terms of timescale and level of action.

### 6.2 Non-invasive mechanical intervention: exploiting “attenuation differences” to turn risk into opportunity

Traditional manual therapies such as massage, deep pressure, stretching, and extracorporeal shock wave therapy (ESWT) have long been used in the treatment of chronic musculoskeletal and fascial pain. In light of the “spatial filter” concept, these interventions can be understood as intentional reconstructions of abnormal mechanical transmission within pathological tissues: by exploiting the features of fibrotic regions—reduced viscoelastic dissipation and “overdeep” force transmission—appropriately parameterized mechanical stimuli may convert otherwise pathogenic mechanical amplification into controlled reparative signals.

During sustained pressure or deep tissue massage, externally applied forces are partly dissipated within superficial fat and fascia, but in high-stiffness structures such as hardened fascial bands and scar cords, impaired stress relaxation leads to more persistent deep traction. When amplitude and duration are appropriately controlled, such moderate-intensity, low-frequency traction can activate Piezo1/TRPV4 pathways on fibroblasts and endothelial cells without strongly activating nociceptive mechanoreceptors, thereby eliciting moderate Ca^2+^ oscillations that promote matrix metalloproteinase upregulation, microcirculatory improvement, and ECM reorganization (Barbe et al., 2021; Gromakovskis, 2025). Studies of fascia and soft tissues have observed that standardized manual therapies improve fascial gliding and local stiffness, reduce trigger-point sensitivity, and relieve pain, findings consistent with this cell–channel–matrix cascade (Barbe et al., 2021; Gehwolf et al., 2025).

ESWT represents another mechanical intervention paradigm characterized by high-energy short pulses plus cavitation. As shock waves propagate through soft tissues, they generate transient high-pressure gradients and local cavitation, producing highly localized, controllable microdamage within fibrotic scars and adhesive bands and thereby disrupting pathologically cross-linked collagen and “cemented” fascial layers (Jin et al., 2025). Unlike extensive tissue disruption, such microdamage typically triggers a repair program dominated by angiogenesis, M2 macrophage polarization, and ECM remodeling, accompanied by scar softening, improved joint mobility, and pain reduction (Jin et al., 2025; Ryskalin et al., 2025).

Clinical systematic reviews have shown that, within reasonable parameter ranges, ESWT significantly improves function and relieves pain in conditions such as plantar fasciitis, tendinopathy, and hypertrophic scars, and that its long-term efficacy often surpasses that of purely analgesic approaches. From a mechanobiological standpoint, ESWT effectively “perforates” hardened barriers and redistributes microscale stress fields, thereby opening new mechanical pathways for subsequent spontaneous remodeling or combined therapies.

### 6.3 The mechanical therapeutic window: biphasic responses and the “just-right” mechanical dose

A growing body of experimental and theoretical work indicates that cellular responses to mechanical stimuli exhibit a characteristic biphasic dose–response relationship: stimuli that are too weak fail to trigger effective remodeling programs, whereas excessively strong stimuli induce overactivation of mechanosensitive channels, Ca^2+^ overload, inflammatory amplification, and even secondary tissue damage. Only within an intermediate range can optimal pro-repair outcomes be achieved. This phenomenon is often referred to as mechanical hormesis (Lin et al., 2026), and the corresponding optimal interval has been vividly described as the “Goldilocks zone.” In the context of the present discussion, this “mechanical therapeutic window” is governed by at least three levels.

At the organ and tissue level, the actual stress–strain field reaching deep cells depends on individual anatomy and fibrosis patterns, and applied surface force alone is an unreliable predictor of deep-tissue stress (Holzapfel et al., 2025; Simonova et al., 2026). At the cellular level, the threshold disparity between reparative cells (fibroblasts, endothelial cells) and pro-pain cells (nociceptive neurons; discussed in Chapter 2), provides a biological basis for selectively engaging repair pathways without eliciting pain (Cheung et al., 2017; Ruviaro et al., 2025). At the clinical level, dose–response data from ESWT, fascial release, and stretching indicate that symptom relief occurs within a relatively narrow parameter band, and machine learning approaches incorporating individual imaging and biomechanical data are beginning to enable individualized treatment recommendations (Sturman and Killingback, 2020; Ryskalin et al., 2025; Chen et al., 2026).

As a starting point toward operationalizing the mechanical therapeutic window, parameters should be defined at the tissue surface where they can be controlled clinically. Applied surface pressure during manual therapy (e.g., massage, sustained compression, stretching) generates low-frequency mechanical loads at frequencies of approximately 1–3 Hz. ESWT operates in a distinct regime, with peak pressures on the order of tens of MPa, microsecond pulse durations, and hundreds to thousands of pulses per session (Ryskalin et al., 2025). As discussed in Chapter 3, these surface loads are substantially attenuated as they propagate through ECM layers, meaning that superficial mechanosensors and deep fibroblasts experience markedly different mechanical signals from the same external input. *In vitro* studies indicate that moderate uniaxial strain can engage reparative programs in fibroblasts via Piezo1/TRPV4-dependent Ca^2+^ signaling, while nociceptive neurons typically require higher or more rapid strain changes for activation (Cheung et al., 2017; Ruviaro et al., 2025). However, the quantitative relationship between a given surface load and the strain experienced by a specific cell at a specific depth in fibrotic tissue has not been systematically calibrated *in vivo*.

Although direct *in vivo* calibration of cellular strain at depth is not yet available, *in vitro* dose–response studies provide provisional quantitative benchmarks. In cultured human dermal fibroblasts subjected to cyclic equibiaxial stretch at 0.1 Hz for 24 h, 10% strain triggered the highest levels of TGF-β1 and collagen expression, whereas 20% strain produced a diminished response. This pattern is consistent with a biphasic dose–response curve in which moderate strain engages reparative programs whereas excessive strain becomes counterproductive (Kuang et al., 2016). In fibroblast-populated collagen gels, collagen accumulation increased exponentially with strain magnitude across the 2%–16% range, with the magnitude and the daily duration of loading independently regulating distinct aspects of matrix remodeling (Balestrini and Billiar, 2009). Conversely, prolonged high-amplitude loading (15% strain at 0.25 Hz for 72 h) induced MMP-2 upregulation without a corresponding increase in type I collagen, shifting the net outcome toward matrix degradation rather than constructive remodeling (Tan et al., 2023). At the single-cell level, Piezo1 activation in cardiac fibroblasts exhibits a force threshold of approximately 300 nN on stiff substrates, and this threshold is substantially reduced on soft substrates, illustrating how ECM stiffness directly modulates the set-point of mechanosensitive channel activation (Braidotti et al., 2024).

These *in vitro* data share a common limitation: they were obtained under defined culture conditions that lack the hierarchical ECM architecture, interstitial fluid flow, and multi-cell-type interactions of intact soft tissues. Consequently, the strain experienced by a fibroblast at a given depth in fibrotic fascia under a given surface load cannot currently be predicted from *in vitro* calibrations alone. The parameter ranges identified are best regarded as testable starting hypotheses for future *in vivo* validation: cyclic strain of 5%–15% at frequencies below 0.5 Hz may be reparative for fibroblasts, whereas sustained or higher-amplitude loading may become pro-fibrotic or catabolic—are therefore best regarded as testable starting hypotheses for future *in vivo* validation, not as clinically actionable prescriptions. Systematic calibration using intravital strain mapping, embedded microscale force transducers, or FRET-based tension sensors (Grashoff et al., 2010), combined with cell-type-specific readouts of Piezo1/TRPV4 activation in healthy versus fibrotic tissues, will be required to translate the mechanical therapeutic window from a conceptual framework into a quantitatively operationalized clinical tool.

Viewed through the lens of the “stiffness–inflammation–pain” closed loop, truly effective strategies do not simply enhance or eliminate mechanical input. Instead, they aim to establish a controllable mechanical therapeutic window among channels, matrix, and external forces. Specifically: first, ECM stiffness and baseline activity of mechanosensitive channels are gradually reduced via soft matrix hydrogels, antifibrotic drugs, and local channel inhibition, partially restoring spatial filtering and stress-relaxation capacity; on this basis, parameters for manual pressure, stretching, and ESWT are optimized using finite element modeling and clinical dose–response data, so that the applied loads remain within the “Goldilocks” interval and preferentially activate repair-related mechanical pathways; finally, once tissue mechanics and neuroimmune status have gradually stabilized, channel inhibitor doses are decreased or withdrawn, allowing the system to reestablish a new steady state at lower gain. This triad of drug–material–mechanical interventions, built around a “window” rather than “extreme suppression” or “extreme stimulation,” represents an important future direction for the refined treatment of chronic soft tissue disorders and related chronic pain.

## 7 Summary and perspectives

Overall, positioning Piezo/TRPV4 as important nodes within the network of soft tissue mechanical state, biochemical signaling, immunity, and neural regulation, and incorporating the spatial filtering function of ECM viscoelasticity into unified consideration, provides a framework that moves beyond traditional linear thinking. Multimodal synergistic strategies centered on the “mechanical therapeutic window”—combining pharmacological intervention, material-based remodeling, and parameterized mechanical therapy—hold promise for more precise treatment of a spectrum of fibrotic diseases and chronic soft-tissue pain.

Several key questions remain open and define important directions for future work. At the structural level, the atomic-resolution binding interfaces between Piezo1 intracellular domains and cytoskeletal tethers such as talin and vinculin await determination. At the tissue level, the quantitative relationship between a given surface mechanical load and the strain experienced by specific cell populations at different depths in fibrotic tissue has not been systematically calibrated *in vivo*, and the predictions of the spatial filter framework require direct experimental testing using tools such as FRET-based tension sensors or intravital strain mapping. At the clinical level, whether the mechanical therapeutic window shifts or narrows with progressive fibrosis, and how to individualize mechanical treatment parameters accordingly, remain unresolved. Addressing these gaps will require interdisciplinary approaches that bridge structural biology, tissue biomechanics, and clinical mechanomedicine, and may ultimately enable therapeutic strategies that are both mechanistically precise and individually optimized.
